# Total cross-sectional area of the femoral neck nutrient foramina measured to assess arterial vascular beds in the femoral head

**DOI:** 10.1186/s13018-019-1480-7

**Published:** 2019-12-13

**Authors:** Jiong Mei, Kun Quan, Hua Wang, Yahui Dai, Fangfang Zhang, Ming Ni

**Affiliations:** 1 Department of Orthopedic Surgery, Shanghai Jiao Tong University Affiliated Sixth People’s Hospital, Shanghai, 200233 China; 2 Department of Orthopedics, The First Affiliated Hospital of Nanchang University, Nanchang, Jiangxi 330006 China; 3 Tongji University School of Medicine, Shanghai, 200092 China; 4 Department of Orthopedics, Songjiang District Central Hospital, Shanghai, 201600 China; 5 Department of Orthopaedics, Pudong New Area Peoples’ Hospital affiliated to Shanghai Health University, Shanghai, 201299 China

**Keywords:** Femoral neck, Femoral head, Nutrient foramina, Three-dimensional computer tomography

## Abstract

**Background:**

A detailed understanding of the blood supply to the femoral head is required to plan the surgery in the femoral neck and head area. However, information about the blood vessel networks in the femoral head is inadequate.

**Methods:**

The surface of the femoral neck of 100 dry cadaveric adult femur specimens was scanned using a 3D scanner. The scanning distance was 200 mm, precision 0.01 mm, and measuring point 0.04 mm. The images were acquired at a resolution of 1,310,000 pixels. Digital imaging data were recorded from the femoral neck surface. The diameters of the nutrient foramina of the superior, inferior and anterior retinacular arteries, and the ligamentum teres arteries were determined and divided into five groups.

**Results:**

The mean cumulative cross-sectional area of the nutrient foramina was as follows: canals of the superior, inferior, anterior, and ligamentum retinacular arteries were 15.59 mm^2^, 3.63 mm^2^, 4.32 mm^2^, and 1.58 mm^2^, respectively. Next, we analyzed the canals of the superior, inferior, anterior and ligamentum retinacular arteries, respectively, via 3D scanner. We found that the canals of the superior retinacular arteries appear to supply more blood to the femoral head than the canals of the other three types of arteries.

**Conclusions:**

Our results demonstrated that surgeries of the femoral neck and femoral head will be improved with prior 3D scanning and lead to better outcomes in surgeries involving the hip area.

## Introduction

The femoral head obtains most of its blood supply from the medial femoral circumflex artery (MFCA) and the lateral femoral circumflex artery (LFCA) [1–4]. A 1953 study reported that the LFCA provides two-thirds to four-fifths of the blood supply to the femoral neck [4]. Traditionally, contrast media has been used to observe the arteries in the area of interest and to measure the retinacular vessels. These arteries branch from the deep femoral artery [5]. The MFCA and LFCA branch to form the retinacular vessels, which enter the capsule and then travel through the retinacula of Weitbrecht, which consists of fibrous extensions from the capsule, and then enter into the nutrient foramina (nutrient artery canals) on the femoral neck, becoming at that point interosseous, and then pass into the femoral head [2, 3, 6]. The retinacular vessels branch from three retinacular arteries: the superior, inferior, and anterior [3]. The first two are from the MFCA and the latter is from the LFCA. Between the MFCA and the LFCA, the MFCA supplies about 80% of the blood to the femoral head; however, in the anteroinferior portion of the femoral head, nearly half of the blood is supplied by the LFCA [2]. In addition to blood from the MFCA and LFCA, the femoral head also receives blood from the round ligament (ligamentum teres) artery [7]. Furthermore, we can observe the number and size of nutrient foramina to determine the blood supply of the femoral neck.

A number of techniques have been employed to measure the arterial vasculature of the femoral head. One method used is the injection of a substance into the MFCA of a cadaver specimen to visualize the arterial system and then follow the course of this system through dissection [3]. In a study to assess the relative contribution of the MFCA and LFCA in supplying blood to the femoral head, both quantitative magnetic resonance imaging (MRI) and computed tomography (CT) have been used [2]. In another study of the arterial blood supply to the femoral head, microCT and three-dimensional (3D) reconstruction were used [4]. Our group used the blunt ends of Kirschner wires for foramina size measurement and CT to obtain a 3D reconstruction of images [8].

The use of 3D scanning to study the arterial vessels that supply blood to the femoral head has some important advantages. This technique allows the storage of information on the surface of the bone. Moreover, it permits the repeated study of the details of the bone surface, which reduces the need for bone specimens.

We previously studied the number and distribution of nutrient foramina in the femoral neck [8]. However, in that study, we did not measure the cross-sectional area of the foramina. Therefore, the aim of this study was to analyze the differential contribution of the sources of blood supply to the femoral neck and head from the major vascular beds and to use a 3D scanner to obtain data on the cross-sectional area of the foramina. It is hoped that furthering knowledge of the blood supply to the femoral neck and femoral head will help surgeons achieve better outcomes in surgeries involving the hip area.

## Methods

### Subjects

A total of 100 dry specimens of adult femurs were obtained from the Department of Anatomy, Tongji University School of Medicine. General information regarding these specimens, such as age, gender, presence of hip disease, and cause of death was unknown.

This study was approved by the Shanghai Tongji Hospital Institutional Review Board.

Specimens were selected according to the following criteria: (1) no significant osteoarthritis or morphological changes within the femoral head; (2) the epiphyseal growth plate of the femoral head was closed; and (3) the femoral neck was intact.

### Measurement of size and number of foramina

The femoral neck surface was scanned using a 3D scanner (Shanghai Digital Manufacturing Corp, Ltd, Shanghai, China). The scanning distance was 200 mm, precision 0.01 mm, and measuring point 0.04 mm. The images were acquired at a resolution of 1,310,000 pixels. Digital imaging data were recorded from the femoral neck surface. Each dry bone specimen was checked to measure the diameter of each nutrient foramen in the digital image. The specific arterial supply to each nutrient foramina was then determined. Since the ligamentum teres arteries are located in a separate area, the place on the nutrient foramina where these arteries entered was easy to identify. To distinguish the nutrient foramina entered by the superior, inferior, and anterior retinacular arteries, we relied on the extensive literature on the anatomy of these arteries [2–4, 6, 9, 10]. The most challenging problem was to identify the edge of each of the three nutrient foramina areas for the canals of retinacular arteries. We defined the edge by the location of the smallest nutrient foramina. Finally, we counted the number of nutrient foramina in each area of the arterial canals.

After 3D imaging, the images obtained from the data were directly outputted into the software by the machine. The images could be freely rotated. Two graduate students measured the diameter of each nutrient foramen in each specimen. The measurements were made while performing a comparison with the corresponding diaphysis specimens to confirm that no measurements were missed. According to the instructions provided, the measured distance between points ranges from 0.04 to 0.08 mm; the maximum acceptable discrepancy was considered to be 0.04 mm. The mean of two measurements was taken as the measurement value in cases where the difference was less than 0.04 mm. When the discrepancy was greater than 0.04 mm, the two graduate students repeated the measurements to obtain mutually consistent values. The quantitative data were measured and quantified using Matic 8.0 software (Materialise Europe, Leuven, Belgium). The measurement data was entered into an Excel spreadsheet (Microsoft Corp., Redmond, WA, USA) for statistical analysis.

### Statistical analysis

All characteristics were summarized as numbers, mean ± standard deviation (SD), and range (min to max). Spearman correlation analysis was performed to identify the correlation between the number and area of total nutrient foramina and the results were shown as coefficients of correlation with corresponding p-values. The total area of nutrient foramina was summarized as mean ± SD (range min to max) for a given size of nutrient foramina at each site. The statistical assessments were two-tailed and considered statistically significant at *p* < 0.05. Data were analyzed using SAS 9.0 (SAS Institute Inc., Cary, NC, USA).

## Results

The characteristics of the 100 bones are presented in Table 1. For all bones, the mean length of the femur was 393.28 mm (SD = 23.88) with a range of 335.60 to 445.89 mm, and the mean length of the femoral neck was 22.53 mm (SD = 3.93) with a range of 15.91 to 35.72 mm. The total number and total area of nutrient foramina were measured for the canals of the superior, inferior, and anterior retinacular arteries and for the ligamentum teres arteries. (Table 1) Of note, 44 samples had no data for the canals of ligamentum teres arteries.

**Table 1:** Summary of the characteristics of the 100 bones

Variables	N	mean±SD	(range: min.-max.)
Length of femur, mm	100	393.28±23.88	(335.6-445.89)
Length of femoral neck. mm	100	22.53±3.93	(15.91-35.72)
Total number of nutrient foramina			
Superior retinacular arteries	100	25.11±6.5	(11-44)
Inferior retinacular arteries	100	5.97±3.25	(1-19)
Anterior retinacular arteries	100	8.23±4.15	(2-22)
Ligamentum teres ateries	56	3.25±2.52	(1-15)
Total -1(included ligamentum teres arteries)	56	42.93±9.31	(22-63)
Total -2 (excluded 1igamentum teres arteries)	44	38.84±9.53	(17-64)
Total area of nutrient foramina. mm^2^			
Superior retinacular arteries	100	15.59±5.71	(5.65-34.27)
Inferior retinacular arteries	100	3.63±2.11	(0.4-11.88)
Anterior retinacular arteries	100	4.32±2.52	(0.79-14.69)
Ligamentum teres arteries	56	1.58±1.21	(0.11-5.43)
Total -1(included ligamentum teres arteries)	56	24.97±6.57	(10.65-42.07)
Total -2 (excluded 1igamentum teres arteries)	44	23.74±7.77	(13.35-43.32)
Ratio of nutrient foramina number in Total			
Superior retinacular arteries / Total-1	56	0.58±0.08	(0.38-0.76)
Inferior retinacular arteries / Total-1	56	0.14±0.06	(0.03-0.33)
Inferior retinacular arteries / Total-1	56	0.20±0.08	(0.07-0.44)
Ligamentum teres arteries / Total-1	56	0.08±0.05	(0.02-0.28)

Correlation analysis showed that the number and cross-sectional area of the nutrient foramina moderately correlated with those of the canals of superior retinacular arteries, anterior retinacular arteries, and ligamentum teres arteries, and weakly correlated with those of the canals of inferior retinacular arteries (*r* = 0.62, 0.80, 0.66, and 0.26, respectively; all *p* values < 0.05) (results not shown in tables or figures).

The ratio of the nutrient foramina number to the nutrient foramina cross-sectional area in each of the four parts of the circulation was also calculated (Table 1). When the canals of ligamentum teres arteries were considered, the mean ratio of the nutrient foramina number (area) was 0.58 (0.60), 0.14 (0.15), 0.20 (0.18), and 0.08 (0.07) in the canals of the superior, inferior and anterior retinacular arteries, and the ligamentum teres arteries, respectively. When the canals of the ligamentum teres arteries were not considered, the mean ratio of the nutrient foramina number (area) was 0.65 (0.68), 0.15 (0.14), and 0.20 (0.17) in the canals of the superior, inferior, and anterior retinacular arteries, respectively. Correlation analysis showed that the ratio of the nutrient foramina number to the nutrient foramina area were all correlated in each portion of the nutrient foramina (canals of ligamentum teres arteries considered: *r* = 0.715, 0.743, 0.839, 0.687 for the canals of the superior, inferior, and anterior retinacular arteries, and the ligamentum teres arteries, respectively; canals of ligamentum teres arteries not considered: *r* = 0.792, 0.718, 0.928 for the canals of the superior, inferior and anterior retinacular arteries, respectively; all *p* value < 0.001) (results not shown in tables or figures).

Table 2 presents the results of the correlation between (1) the length of the femur and the number of nutrient foramina, (2) the length of the femur and the area of the nutrient foramina, (3) the length of the femoral neck and the number of nutrient foramina, and (4) the length of the femoral neck and the area of the nutrient foramina, for the canals of the retinacular arteries and the ligamentum teres arteries. The results showed that the number of nutrient foramina for the canals of the superior retinacular arteries was significantly positively correlated with the length of the femur (*r* = 0.219), the area of the nutrient foramina for the canals of the superior retinacular arteries was significantly positively correlated with the length of the femur and the length of the femoral neck (*r* = 0.280 and 0.243), and the area of the nutrient foramina for the canals of the inferior retinacular arteries was significantly positively correlated with the length of the femoral neck (*r* = 0.207) (Table 2).

**Table 2:** Correlation of the length of the femur, length of the femoral neck, and nutrient foramina at each site

Site of length	Measured type of nutrient foramina	Site of nutrient foramina			
		Canals of superior retinacular arteries	Canals of inferior retinacular arteries	Canals of anterior retinacular arteries	Canals of ligamentum teres arteries
Femur	number	0.219*	0.079	-0.122	-0.066
	area	0.280**	0.130	-0.097	-0.044
Femoral neck	number	-0.020	0.130	-0.013	0.122
	area	0.243*	0.207*	0.150	0.195

Results are presented as coefficient of correlation

*p<0.05, **p<0.01, indicate significant difference (*p* value < 0.05)

**Fig 1:**
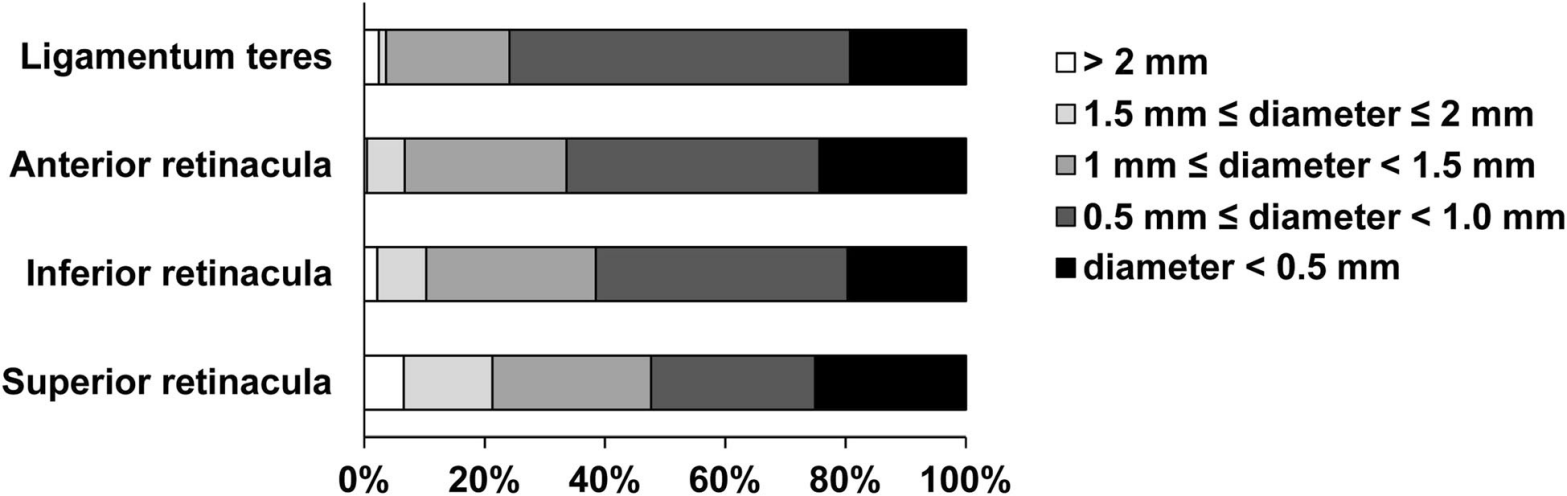
Dispersion of the percentage of samples for measuring the nutrient foramina by stratification of the size of nutrient foramina at different sites

In this study, all samples were measured more than once. There was a total of 922 measurements of the nutrient foramina of the 100 samples: 40% (367/922) for the canals of the superior retinacular arteries, 26% (234/922) for the canals of the inferior retinacular arteries, 25% (238/922) for the canals of the anterior retinacular arteries, and 9% (83/922) for the canals of the ligamentum teres arteries (Additional file 1: Table S1). Furthermore, the nutrient foramina measurements of the different sites were stratified by the size of the nutrient foramina as shown in Fig. 1. The total area of the nutrient foramina for the four major vascular beds of the femoral head was stratified by the size of the nutrient foramina into five groups (Table 3). Importantly, most of the nutrient foramina (> 50%) had a diameter of less than 2 mm at each site. The majority of nutrient foramina had a diameter of 0.5 to 1.0 mm in each group. However, foramina with a diameter 1.0 to 1.5 mm and less than 0.5 mm were abundant for the canals of the superior retinacular arteries. Only a few nutrient foramina of the canals of the ligamentum teres arteries had a diameter greater than 1.5 mm. The mean area was focused on arteries with a diameter of 0.5 to 1.5 mm. Although not many nutrient foramina had a diameter greater than 2.0 mm, the total area of these foramina was not small.

**Table 3:** Summary of the total area of the nutrient foramina for given size of nutrient foramina at each site

Size of nutrient foramina	Superior retinacular arteries			Inferior retinacular arteries			Anterior retinacular arteries			Ligamentum teres arteries		
	n	mean±SD	range (min., max.)	n	mean±SD	range (min., max.)	n	mean±SD	range (min., max.)	n	mean±SD	range (min., max.)
D > 2 mm	24	4.47 ± 1.69	(3.2 , 9.75)	5	4.04 ± 0.86	(3.3 , 5.51)	1	5.51	-	2	4.07 ± 0.43	(3.76 , 4.37)
1.5 mm ≤ D ≤ 2 mm	54	3.73 ± 1.98	(1.77 , 10.11)	19	2.34 ± 0.35	(1.84 , 3.08)	15	2.3 ± 0.58	(1.77 , 3.85)	1	1.77	-
1 mm ≤ D < 1.5 mm	97	5.09 ± 3.1	(0.82 , 15.2)	66	1.73 ± 0.94	(0.8 , 5.88)	64	2.13 ± 1.26	(0.8 , 5.49)	17	1.24 ± 0.42	(0.83 , 2.17)
0.5 mm ≤ D < 1.0 mm	100	7.07 ± 2.61	(1.81 , 18.22)	98	1.8 ± 1.06	(0.2 , 5.83)	100	2.4 ± 1.29	(0.31 , 7.02)	47	1.14 ± 0.91	(0.28 , 5.09)
D < 0.5 mm	92	0.54 ± 0.37	(0.07 , 2.01)	46	0.19 ± 0.14	(0.07 , 0.87)	58	0.28 ± 0.19	(0.07 , 1.04)	16	0.24 ± 0.16	(0.08 , 0.69)

Abbreviations: D, diameter; SD, standard deviations; min., minimum; max., maximum

## Discussion

**Fig 2:**
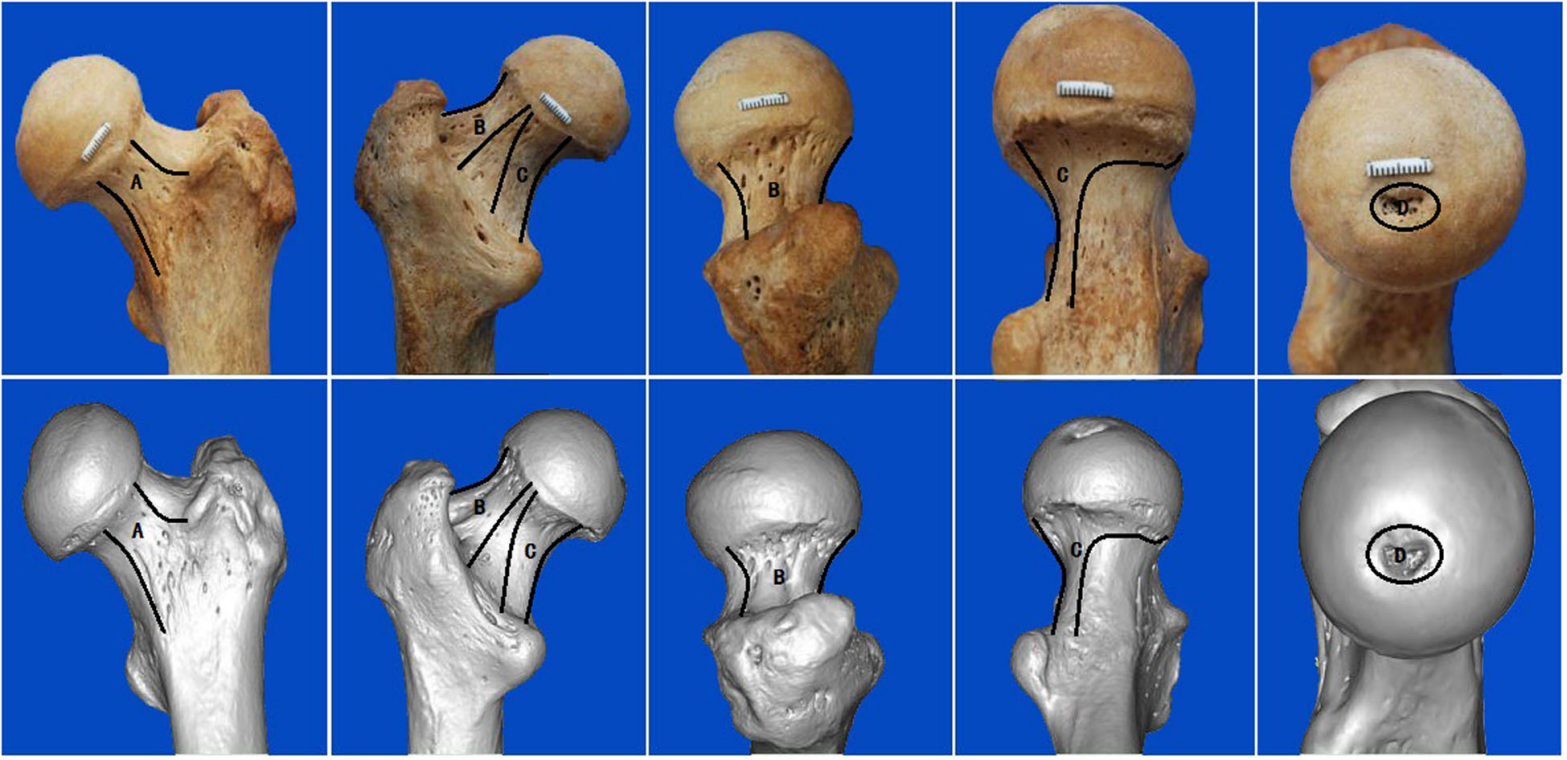
3D scanning images that allow the precise restoration of the observed object. Images of the femoral head and neck and the posterior, anterior, upper, and lower surfaces can be seen. The lower row shows images taken using a 3D scanner, and the order of the images corresponds to that of the images taken using a camera in the upper row. The images in the two rows reveal a high degree of consistency in the nutrient foramina. **a** Anterior retinacular. **b** Superior retinacular. **c** Inferior retinacular. **d** Ligamentum teres

In this study, we investigated the vascular blood supply to the femoral neck and femoral head by using a 3D scanner to precisely measure the cross-sectional area of the nutrient foramina in the femoral neck of dry adult femur specimens. This method allows precise restoration of the scanned object (Fig. 2). Data were obtained for the four major vascular canals, the superior, inferior, and anterior retinacular arteries, and the ligamentum teres arteries. The mean total cross-sectional area was largest for the canals of the superior retinacular arteries and smallest for the canals of the ligamentum teres arteries; in 44 of the 100 samples, there was no data for the canals of the ligamentum teres arteries. The data we obtained should be useful for surgeons performing hip surgery, as greater understanding of the details of the vascular blood supply to the femoral neck and femoral head should help improve outcomes.

The method we used to measure the cross-sectional area of the foramina in the femoral neck to assess the relative contribution of various blood vessels to the blood supply of the femoral head may have provided more objectivity for understanding the vascular blood supply of the femoral head than the methods used in previous studies. This difference may explain why some other studies found arteries other than the superior retinacular arteries to be the most important for supplying blood to the femoral head. More than 60 years ago, Trueta et al. [4] reported that the lateral epiphyseal arteries are the main providers of blood to the femoral head. However, their method was somewhat subjective, being based on interpretation of perfusion radiographs. Lazaro et al. [3] perfused the MFCA in cadaveric specimens and followed the course of the artery by dissection. They reported that blood vessels of the inferior retinaculum on the inner side of the femoral neck provide approximately 30% of the blood supply to the femoral head. Zhao et al. [10], in a study that used angiographic methods and microCT scans to reconstruct 3D structures, concluded that the inferior retinacular arteries are essential to the blood supply of the femoral head. The method we used in this study may be more objective than perfusion methods for the following reasons: (1) nutrient foramina are the only channels through which peripheral blood can enter bones; (2) the cross-sectional area of the nutrient foramina can reflect the thickness of the blood vessels passing through them, and therefore reflect the amount of blood flow; (3) using images acquired with a 3D scanner allows for precise measurements of the diameter of the nutrient foramina, which permits calculation of the total cross-sectional area of the region observed, thereby providing more objectivity than perfusion methods; and (4) all blood vessels pass through the nutrient foramina anastomose within the bone.

Sevitt et al. [9] reported that, when perfusion of only the round ligament was performed in 17 specimens, the branches of the artery did not enter the femoral head in 6 specimens. The vicinity of the pit in the femoral head had perfusion in 6 specimens and other areas of the femoral head had perfusion in 5 specimens (the outer side or lower portion of the femoral head); the entire femoral head received perfusion in only 1 specimen. However, Zhao et al. [10] recently reported that the inferior retinacular arterial system appears to be the important structure maintaining the femoral head blood supply after femoral neck fracture. Although this could be true, it should be pointed out that Zhao et al. did not consider individual differences in vascular distribution.

**Fig 3:**
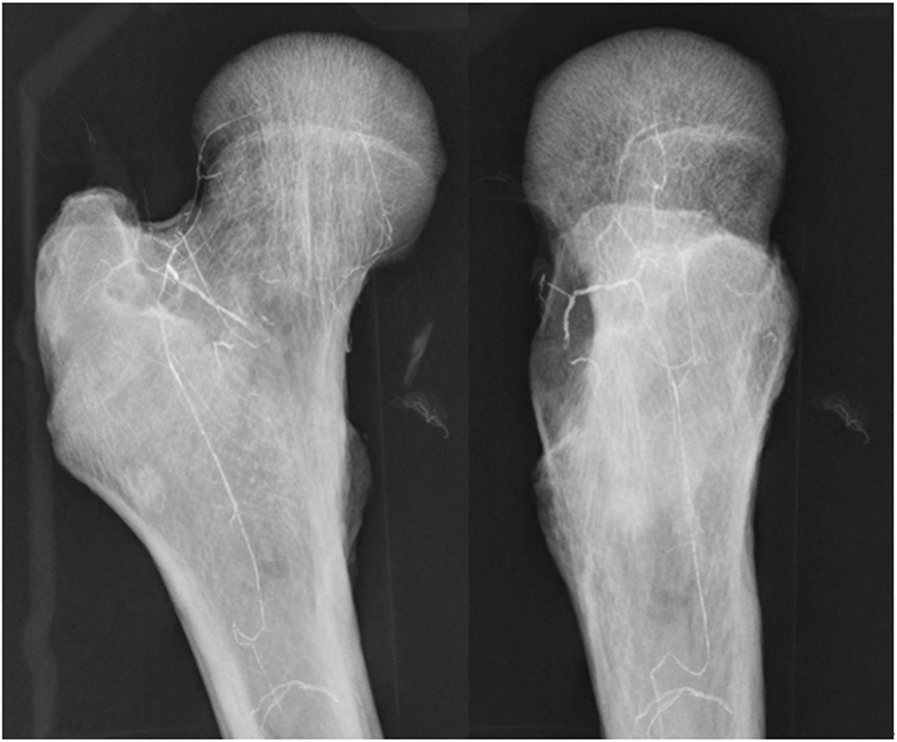
Perfusion imaging of the ascending branch of the femoral trophoblastic arteries. Perfusion imaging after ligation of the internal and external arteries at the origin of the deep femoral artery revealed that the ascending branch of the nutrient artery of the femoral shaft follows the medullary cavity of the femoral shaft, and that superior and inferior retinacular arteries communicate at the base of the femoral neck and at the femoral neck, and ultimately extend within the femoral head

Previous studies found that the ascending branch of the nutrient artery of the femoral shaft ascends following the medullary cavity of the shaft, and generally only reaches the base of the femoral neck. However, when we carried out perfusion imaging of a fresh cadaveric specimen, we initially ligated the internal and external arteries at the origin of the deep femoral artery and severed the round ligament prior to performing perfusion imaging (data not shown). We observed that the upper superior and lower inferior retinacular arteries communicate at the base of the femoral neck, and ultimately extend within the femoral head. This result provided verification that the ascending branch of the nutrient artery of the femoral shaft can also reach the femoral head, passing through the anastomoses in the bone (Fig. 3). This observation has not been previously mentioned in the literature.

In our recent study of the number and distribution of nutrient foramina in the femoral neck, we noted that “Precise knowledge of the vascular supply to the femoral head is critical when contemplating surgery in the region surrounding the femoral head and neck junction” [7]. This is applicable both to the bleeding per se related to surgery and to the sequelae. The extent of bleeding of the femoral head in total hip arthroplasty for osteoarthritis has been studied by Schwartsmann et al. [11]. In the treatment of femoral hip fractures, the sequelae of most concern are femoral neck nonunion and avascular necrosis [12]. These sequelae are also of great concern in hip resurfacing in patients with hip osteoarthritis [13].

The aberrant arteries and the numerous anatomical variations of vessels represent some important risks during medical imaging and surgical procedures, and specialists must know these unusual aspects and topographies in daily practice [14]. A recent study by Rego et al. [15] was carried out to investigate the arterial anatomy in the region of the femoral head-neck perforation. Using fresh proximal parts of the femur and MRI with the injection of gadolinium, this study found that the mean number of arterial foramina was 4.5 and the foramina were predominantly located between 10 and 12 o’clock. The study design eliminated the possibility that the foramina were exclusively for veins. The authors speculated that the much higher number of foramina reported in dry bone studies might be attributable to a majority of foramina corresponding to venous vessels. The difference in findings with regard to the number of foramina in dry bone studies such as ours and studies using fresh bone requires further exploration.

This study had limitations. The blood supply to the femoral head was not obtained by direct measurements, but indirectly by calculating the number and cross-sectional area of the nutrient foramina. More direct measurements of blood supply are needed to confirm our results. We used a 3D scanner that is used commercially to inspect holes in jewelry rather than the 3D CT scanner used clinically. However, since the nutrient foramina are larger than the holes in gems, we are confident of having obtained precise measurements of the foramina.

To summarize, the results of this study extend the knowledge about the blood supply to the femoral neck and femoral head. By using an objective method to precisely measure the cross-sectional area of the nutrient foramina in the femoral neck, we could more clearly define the contributions of the four major vascular beds, the superior, inferior and anterior retinacular arteries, and the ligamentum teres arteries, than has been achieved in previous research efforts. Based on the total cross-sectional area of the nutrient foramina, our results indicate that the superior retinacular arteries supply the most blood to the femoral head. Improved understanding of the blood supply to the femoral head should help surgeons obtain better results when performing surgery in the hip area.

## Supplementary information

**Additional file 1: Table S1.** Summary of the frequency of nutrient foramina for given size of nutrient foramina at each site.

## Data Availability

Not applicable.
