# Supplementary material for: Total cross-sectional area of the femoral neck nutrient foramina measured to assess arterial vascular beds in the femoral head
Source: J Orthop Surg Res. 2019 Dec 13;14:439. doi: 10.1186/s13018-019-1480-7 (PMC6911289; doi:10.1186/s13018-019-1480-7)
Supplement: Supplementary file 1 — Additional file 1: Table S1. Summary of the frequency of nutrient foramina for given size of nutrient foramina at each site. [file 13018_2019_1480_MOESM1_ESM.docx]

**Table S1.** Summary of the frequency of nutrient foramina for given size of nutrient foramina at each site

| **Size of nutrient foramina** | **Number of nutrient foramina** | **Superior retinacular arteries  (Number of samples)** | **Inferior retinacular arteries  (Number of samples)** | **Anterior retinacular arteries  (Number of samples)** | **Ligamentum teres arteries  (Number of samples)** |
| --- | --- | --- | --- | --- | --- |
| Total |  | 367 | 234 | 238 | 83 |
| diameter > 2 mm |  |  |  |  |  |
|  | 1 | 20 | 5 | 1 | 2 |
|  | 2 | 4 |  |  |  |
|  | Total | 24 | 5 | 1 | 2 |
| 1.5 mm ≤ diameter ≤2 mm |  |  |  |  |  |
|  | 1 | 30 | 19 | 14 | 1 |
|  | 2 | 18 |  | 1 |  |
|  | 3 | 3 |  |  |  |
|  | 4 | 3 |  |  |  |
|  | Total | 54 | 19 | 15 | 1 |
| 1 mm ≤ diameter <1.5 mm |  |  |  |  |  |
|  | 1 | 12 | 39 | 33 | 14 |
|  | 2 | 15 | 19 | 14 | 3 |
|  | 3 | 16 | 7 | 8 |  |
|  | 4 | 13 | 1 | 8 |  |
|  | 5 | 11 |  | 1 |  |
|  | 6 | 10 |  |  |  |
|  | 7 | 8 |  |  |  |
|  | 8 | 3 |  |  |  |
|  | 9 | 5 |  |  |  |
|  | 10 | 2 |  |  |  |
|  | 13 | 2 |  |  |  |
|  | Total | 97 | 66 | 64 | 17 |
| 0.5 mm ≤ diameter <1.0 mm |  |  |  |  |  |
|  | 1 | 0 | 13 | 1 | 13 |
|  | 2 | 0 | 11 | 14 | 16 |
|  | 3 | 1 | 17 | 10 | 6 |
|  | 4 | 0 | 22 | 10 | 4 |
|  | 5 | 0 | 16 | 14 | 5 |
|  | 6 | 2 | 6 | 18 | 1 |
|  | 7 | 1 | 4 | 12 | 0 |
|  | 8 | 2 | 2 | 6 | 0 |
|  | 9 | 4 | 3 | 5 | 1 |
|  | 10 | 7 | 1 | 3 | 0 |
|  | 11 | 6 | 2 | 1 | 0 |
|  | 12 | 3 |  | 2 | 0 |
|  | 13 | 4 |  | 2 | 1 |
|  | 14 | 10 | 1 | 1 |  |
|  | 15 | 6 |  |  |  |
|  | 16 | 9 |  |  |  |
|  | 17 | 8 |  | 1 |  |
|  | 18 | 6 |  |  |  |
|  | 19 | 6 |  |  |  |
|  | 20 | 1 |  |  |  |
|  | 21 | 6 |  |  |  |
|  | 22 | 5 |  |  |  |
|  | 23 | 2 |  |  |  |
|  | 24 | 2 |  |  |  |
|  | 25 | 2 |  |  |  |
|  | 26 | 2 |  |  |  |
|  | 28 | 1 |  |  |  |
|  | 29 | 2 |  |  |  |
|  | 30 | 1 |  |  |  |
|  | 35 | 1 |  |  |  |
|  | Total | 100 | 98 | 100 | 47 |
| diameter <0.5 mm |  |  |  |  |  |
|  | 1 | 14 | 36 | 30 | 8 |
|  | 2 | 22 | 8 | 18 | 6 |
|  | 3 | 21 |  | 5 | 1 |
|  | 4 | 7 |  | 1 | 1 |
|  | 5 | 12 |  | 3 |  |
|  | 6 | 8 | 2 |  |  |
|  | 7 | 2 |  |  |  |
|  | 8 | 1 |  | 1 |  |
|  | 9 | 2 |  |  |  |
|  | 10 | 1 |  |  |  |
|  | 13 | 1 |  |  |  |
|  | 14 | 1 |  |  |  |
|  | Total | 92 | 46 | 58 | 16 |

Results are a summary of the number of samples (one sample may be used more than once).
